# The Elect, or Eugenics and Woman Suffrage

**Published:** 1913-05

**Authors:** 


### Department of Eugenics.

Conducted by Dr. Malone Duggan and Dr. Theo. Y. Hull.

### THE TEXAS STATE SOCIETY OF SOCIAL HYGIENE.

Headquarters: San Antonio, Texas.

DR. MALONE DUGGAN, President, San Antonio.
DR. F. E. DANIEL, 1st Vice President, Austin.
PROF. A. CASWELL ELLIS, 2d Vice President, Austin.
MRS. ELI HERTZBERG, 3d Vice President, San Antonio.
DR. THEO. Y. HULL, Secretary, San Antonio.
MR. W. L. EVANS, Treasurer, San Antonio.

EXECUTIVE COMMITTEE.

| | | |
|---|---|---|
| Dr. Malone Duggan, Chm. | Hon. J. C. Willacy. | Dr. W. A. King. |
| Dr. Theo. Y. Hull, Sec'y. | Mr. W. L. Evans. | Dr. J. W. Torbett |
| Dr. W. F. McCaleb. | Dr. Frank D. Boyd. | Dr. F. C. Walsh. |
| Hon. C. H. Jenkins. | Dr. J. R. Nichols. | Dr. Joe Reuss. |
| Rev. A. W. S. Garden. | Dr. J. M. McCutchan. | Dr. Frank Paschal. |

### The Elect, or Eugenics and Woman Suffrage.

The business of eugenics is race culture. The success of race culture depends upon the character of the offspring. The character of the offspring is determined by the hereditary units of the parents. These units are only affected by influences that modify the germinal cells. These influences are most operative before, during and after gestation. Therefore, woman is the most important factor in generation, and eugenics must give her the first consideration in its program. Woman suffrage concerns eugenics only to the extent of the effect that the movement will have on maternity. If exercise of the ballot will assure a higher standard for motherhood, then the ballot is right. But if it is in any way detrimental to the maternal interest of the mother it is all wrong. This is a plain statement of facts, but it is the supreme test to any question affecting the welfare of woman.

Then we ask the question, Will the ballot have any deterrent effect on maternity? The eugenist's reply is, It will. His reason is based upon the genetic difference between the sexes, determining a definite and distinct purpose for both sexes and making one the complement of the other. This difference premises the necessity for distinct treatment of the two, to the end that the individual characteristics are developed and maintained.

Suffrage, then, is not a question of equality, or superiority, or moral reform but whether or not it will benefit or deter the highest purpose of nature.

The mere registration of a vote or expression of opinion could not conceivably make the slightest difference in the individuality. But the exercise of the ballot does not stop there. The sex difference is particularly marked in the peculiar psychological temperament of woman. She is more conscientious, more emotional, more enthusiastic, more changeable and more vehement than man, and voting will excite these emotions in an inordinate degree, to the sacrifice of her maternal instincts and innate refinement. Again, the lure of politics will drag woman more and more into competition with the business of man. This constant friction is most destructive to her femininity, and will be at the sacrifice of chivalry. Consequently, with her most distinctive characteristic blunted and warped, woman will have little protection against the superior force of masculine inhumanity.

The eugenist believes that society has entirely mistaken this "great unrest," that has thrashed itself into militant action, as due to the inequality of political rights, whereas the trouble lies in a deeper and more personal problem. Since the awakening of the new sex dispensation it has gradually dawned upon society that woman has not been justly dealt with. She has attributed her sex aberrations and impulses to other causes and has accepted the dictates of society on the moral law as final. This inequality and subjugation of her higher innate principles rightfully protest, and it is the aim of eugenics to adjust, so far as possible, these differences on their proper basis.

Respect, admiration, love and perfect freedom in sexual selection; compatibility of temperament and a due regard for the responsibility of parenthood. The development of a normal sexual instinct, sublimated by intellectual refinement and force of character. Equality before the moral law and release from sex subjugation by divorce. When woman achieves this freedom of personal action, the question of woman's rights will be solved.

When suffrage has obtained—for "it is coming"—the militant army dispersed and the conquering hosts returned to their homes; when the smoke of battle has cleared away, and ruffled feelings soothed to peaceful repose; when society has once more found its sea legs, there will be a wonderful revelation. The woman who *knew* and chose rather to be true to her nature and exalt motherhood and womanhood, will be ensconced in the citadels of the nation, loved and adored, the wife, the mother, the arbiter of human destiny, happy and content—*the elect.*

